# Small “Nested” Introgressions from Wild *Thinopyrum* Species, Conferring Effective Resistance to Fusarium Diseases, Positively Impact Durum Wheat Yield Potential

**DOI:** 10.3390/plants10030579

**Published:** 2021-03-19

**Authors:** Ljiljana Kuzmanović, Gloria Giovenali, Roberto Ruggeri, Francesco Rossini, Carla Ceoloni

**Affiliations:** Department of Agriculture and Forestry Science, University of Tuscia, 01100 Viterbo, Italy; gloria.giovenali@unitus.it (G.G.); r.ruggeri@unitus.it (R.R.); rossini@unitus.it (F.R.); ceoloni@unitus.it (C.C.)

**Keywords:** chromosome engineering, breeding, pre-breeding, alien introgression, homoeologous recombination, grain number, adaptability, gene pyramiding

## Abstract

Today wheat cultivation is facing rapidly changing climate scenarios and yield instability, aggravated by the spreading of severe diseases such as Fusarium head blight (FHB) and Fusarium crown rot (FCR). To obtain productive genotypes resilient to stress pressure, smart breeding approaches must be envisaged, including the exploitation of wild relatives. Here we report on the assessment of the breeding potential of six durum wheat-*Thinopyrum* spp. recombinant lines (RLs) obtained through chromosome engineering. They are characterized by having 23% or 28% of their 7AL chromosome arm replaced by a “nested” alien segment, composed of homoeologous group 7 chromosome fractions from *Th. ponticum* and *Th. elongatum* (=7el_1_L + 7EL) or from different *Th. ponticum* accessions (=7el_1_L + 7el_2_L). In addition to the 7el_1_L genes *Lr19 + Yp* (leaf rust resistance, and yellow pigment content, respectively), these recombinant lines (RLs) possess a highly effective QTL for resistance to FHB and FCR within their 7el_2_L or 7EL portion. The RLs, their null segregants and well-adapted and productive durum wheat cultivars were evaluated for 16 yield-related traits over two seasons under rainfed and irrigated conditions. The absence of yield penalties and excellent genetic stability of RLs was revealed in the presence of all the alien segment combinations. Both 7el_2_L and 7EL stacked introgressions had positive impacts on source and sink yield traits, as well as on the overall performance of RLs in conditions of reduced water availability. The four “nested” RLs tested in 2020 were among the top five yielders, overall representing good candidates to be employed in breeding programs to enhance crop security and safety.

## 1. Introduction

Durum wheat (*Triticum durum* Desf., 2n = 4x = 28, genomes AB) is cultivated on only 8% of the global land surface planted to wheat [1,2], yet it is a strategic crop for countries across diversified world areas, primarily in the Mediterranean Basin [3,4]. By domestication and historical selection for a limited number of traits, the original genetic variability present in durum wheat’s direct progenitors and landraces was significantly restricted [5]. This was one of the causes of slowing down and stagnation of yield increase rates [6,7]. In sharp contrast with the current scenario, global wheat production should increase 1.8% per year to double by 2050 and thus secure sufficient supply for the growing world population [8]. Taking into consideration also the ongoing climate changes, wheat breeding today has to accomplish the hard task of providing good yielding varieties with the capacity of adapting to and coping with new abiotic and biotic stresses [9]. Climate changes not only contributed to modify the durum wheat conventional distribution areas and to increase the differential response of genotypes to environments by 49% since the mid-1980′s [10], but also exposed the crop to unfamiliar pathogens [11,12]. This is the case for fungal pathogens of the *Fusarium* genus, responsible for some of the most threatening diseases of wheat and other cereals, namely Fusarium head blight (FHB) and Fusarium crown rot (FCR). As a consequence of global warming, anthesis occurs earlier during the growth cycle, which creates a more favourable timing for flower-infecting fungi, like FHB causal agents (e.g., [13,14]). Likewise, ever-increasing water-deficit and hot conditions during flowering and grain-filling stages, as in semi-arid world regions, contribute to boosting FCR incidence and severity [15]. For the tetraploid durum wheat, which is more vulnerable to both FHB and FCR than the hexaploid bread wheat (*T. aestivum* L., 2n = 6x = 42, ABD; reviewed in [16]), this condition is particularly worrying. Typically intended for human consumption, the durum wheat crop greatly suffers, besides yield and quality reduction, the safety problems associated with health-dangerous *Fusarium* mycotoxins, such as deoxynivalenol [17].

The selection of better varieties greatly depends on the identification of new genetic variability. On one side, recent developments in sequencing technologies and haplotype analyses have shown that even within cultivated wheat germplasm, considered to have a restricted genetic base, there is still a significant amount of unexploited alleles and their combinations for breeding [18,19,20]. On the other hand, exotic genomes of wheat wild relatives represent a much richer source of genetic diversity, still largely unexplored and little exploited (e.g., [21,22,23,24]). The transfer of wild genes into cultivated wheats to make them useful for agriculture is not routinely employed yet, due to the existence of interspecific barriers, time-requiring steps of selection, accurate phenotyping of target products and recurrent backcrossing to adapted genotypes, and not least, a scarcity of adequate expert competences. Nevertheless, some advanced options that can speed up the alien-to-wheat gene transfer have been developed, such as chromosome engineering [25,26], a validated and sustainable cytogenetic approach successfully applied to both bread and durum wheat as recipient species (e.g., [11,27,28]). This is a smart strategy that is able to deliver products of homoeologous recombination, i.e., wheat genotypes containing chromosome segments from related Triticeae species of highly similar gene content. As a result, such alien segments can largely compensate for the replaced wheat counterparts. Germplasm obtained by these means represents ideal material for precision phenotyping and functional dissection of wild chromosome introgressions (e.g., [29,30]). An accurate characterization of alien genetic content is necessary for proper evaluation of its potential for agriculture. To do this, the integration of genotyping with high-precision phenotyping is a new paradigm in today’s breeding, which increases the probability of leveraging cumulative gene effects underlying quantitative traits such as yield [31].

In most cases when chromosome engineering is used, only one wild species is targeted as the donor of a chromosome segment. Successful examples of introgression from multiple alien donors into wheat are very few, particularly when the less tolerant tetraploid durum wheat is involved (e.g., [28]). Noteworthy cases include the development of bread and durum wheat recombinant lines containing single alien segments on the long (L) arm of chromosome 7D, or 7A, respectively, composed of genetic material from decaploid *Thinopyrum ponticum* (Popd.) Barkworth and D.R. Dewey (2n = 10x = 70, genomes E^e^E^b^E^x^StSt) and diploid *Th. elongatum* (Host) D.R. Dewey (2n = 2x = 14, genome E) [32,33]. Through this strategy, useful genes from two alien genomes were pyramided within a single small-sized chromosome segment. Specifically, *Lr19*, a still highly effective leaf rust resistance gene, *Yp*, increasing semolina yellow pigmentation, and yield-contributing loci (see ahead) from *Th. ponticum* accession el_1_ were combined with either one of two major QTL for resistance to FHB and FCR, here referred to as *Fhb7el*_2_ (from *Th. ponticum* accession el_2_) or *Fhb7E* (from *Th. elongatum*). The two FHB/FCR resistance QTL are likely orthologs on 7el_2_L and 7EL arms ([32,33,34,35], and references therein).

Durum wheat recombinants with composite 7el_1_L + 7el_2_L or 7el_1_L + 7EL introgressions were obtained by nesting the 7el_2_L or 7EL most distal portions within 7el_1_L segments present in previously produced durum wheat recombinants R5 and R112 (23% and 28% of their 7AL arms, respectively, replaced by 7el_1_L; [36]). As donor of the *Fhb7el*_2_ QTL, the 7DS·7el_2_L centric bread wheat-*Th. ponticum* translocation line was used [32], whereas bread wheat 7DS·7DL-7el_1_L/7EL recombinant types (named R69-9 and R74-10), obtained in an earlier transfer [34], were employed as source of the *Fhb7E* QTL. Nesting of both *Fhb7* QTL was achieved through homologous pairing and recombination, between 7el_1_L and 7el_2_L for *Fhb7el*_2_, and between the 7el_1_L portions shared by donor and recipient recombinant chromosomes for *Fhb7E*. When challenged with *F. graminearum* spike inoculation, 7el_1_L + 7el_2_L and 7el_1_L + 7EL recombinants exhibited an average of 75%, and >90%, respectively, reduction in FHB severity [32,33]. Moreover, unlike the case of *Fusarium* spp. resistance QTL native to wheat, both *Fhb7el*_2_ and *Fhb7E* were shown to confer tolerance to FCR as well, with disease index of *F. culmorum* inoculated seedlings reduced by over 50% ([33] and unpublished).

The stacking of effective resistance to both FHB and FCR with positive attributes of 7el_1_L derivation can markedly increase the breeding value of primary recombinants R5 and R112. The effect on yield and yield-related traits of 7el_1_L segments present in R5 and R112 was extensively analysed in multi-year and multi-location field trials across three continents [29,37,38,39,40,41]. Several key yield-contributing traits were enhanced by the presence of specific fractions of the alien segments, e.g., spikelet number/spike and spike index by the 23%-long 7el_1_L segment of R5, or tiller number, flag leaf area, chlorophyll content, biomass and grain yield by the 23–28% 7el_1_L portion specific to R112. As to the secondary recombinants derived from R5 and R112, harbouring the composite 7el_1_L + 7el_2_L or 7el_1_L + 7EL segments, preliminary assessments of early generations after the transfer from the respective bread wheat parents provided with encouraging outcomes [32,33]. However, larger scale field tests of more advanced generations in different environmental conditions are needed for more sound evaluation of the impact on yield of the novel alien segment assemblies. Therefore, the aim of the present work was to carry out a more detailed appraisal of the yield potential of all the different secondary recombinants (7el_1_L + 7el_2_L and 7el_1_ + 7EL, with 7el_1_L being of the R5 and R112 type), in order to provide sound evidence of the possibility to effectively exploit such wheat-alien introgression lines in breeding. In this view, possible genetic effects of the nested 7el_2_L and 7EL segments, as compared to those of the original 7el_1_ segments, were analysed, and the field performance of all recombinants assessed in comparison with widely cultivated durum wheat varieties.

## 2. Materials and Methods

### 2.1. Plant Material

**Figure 1:**
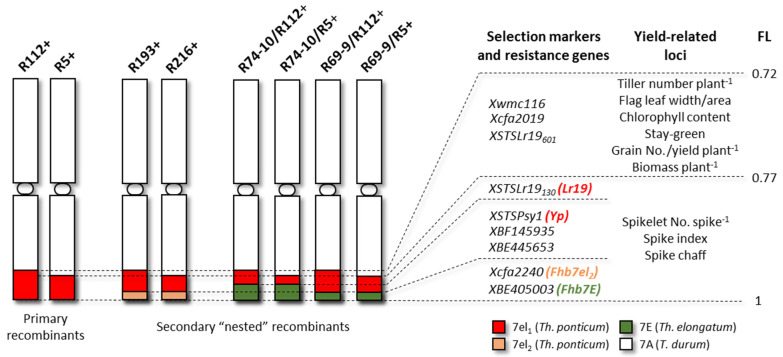
Cytogenetic maps of 7A chromosomes of recombinant lines and loci for yield-related traits, associated with *Th. ponticum* 7el_1_L segments of primary recombinants (adapted from [29,37]). Resistance genes are indicated in the colour of wild species of origin; FL, fractional arm length of the distance from centromere (0) to telomere (1).

**Figure 2:**
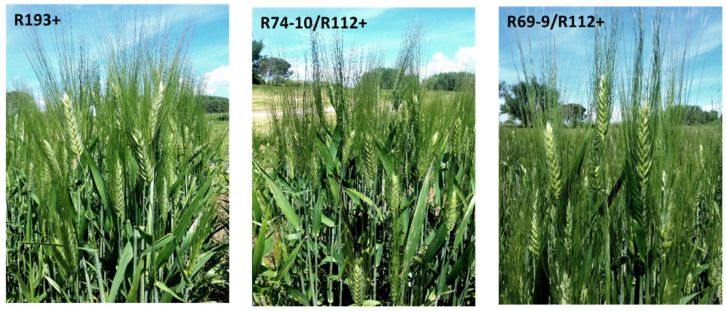
Field view of the R112+ derived nested recombinant lines in 2019 season.

Durum wheat-*Thinopyrum* spp. recombinant lines (RLs), which are homozygous carriers (hom+) of a given alien segment on the wheat 7AL arm, and the respective non-carrier (hom−) lines were analysed in the present study. Their distinctive features are reported in Figure 1 and Table 1. The set of hom+ lines comprises two primary recombinants, R5+ and R112+, harbouring 7el_1_L segments from *Th. ponticum* accession el_1_ [36] in a near-isogenic background of cv. Simeto, and six secondary recombinants, containing combined 7el_1_L + 7el_2_L or 7el_1_ + 7EL alien segments. The 7el_2_L and 7EL segments, deriving from *Th. ponticum* accession el_2_ and *Th. elongatum*, respectively, are “nested” within the primary 7el_1_L segments (see Introduction). R5+ and its derivatives (R216+, R74-10/R5+, R69-9/R5+) have 23% of the 7AL arm replaced by their alien introgression, while R112+ and its derivatives (R193+, R74-10/R112+, R69-9/R112+, Figure 2) have their alien segment occupying 28% of the 7AL arm (Figure 1). Based on cytogenetic maps [32,33,34], the size of nested 7el_2_L and 7EL segments is are similar, with the breakpoint along 7el_1_L being distal to the *Yp* locus in all cases (hence *Lr19* always retained), except for R74-10 derivatives (Figure 1). For each secondary hom+ line, a corresponding hom− line, isolated from the same segregating progeny, was available and used here as the negative control in the assessment of the effect of a given alien segment on the measured traits. The following control lines were also included: (i) Italian cultivar Simeto and the ICARDA elite genotype IDYT22 (=Secondroue, kindly provided by Dr Filippo Bassi, ICARDA), included in the RLs’ backgrounds (Table 1), both well-adapted to drought-prone environments and characterized by high grain weight, with IDYT22 being also moderately resistant to yellow and leaf rust as well as to Septoria tritici blotch; (ii) four of the best yielders among the currently and widely cultivated Italian durum varieties, namely Monastir, Ettore, Kanakis and Antalis, well-adapted to Central Italy growing conditions [42], where the present experiments were conducted. Interestingly, they are also among the best performers under no-till cultivation in hot and dry environments of insular and peninsular Italy [43].

### 2.2. Field Trials and Trait Measurements

**Figure 3:**
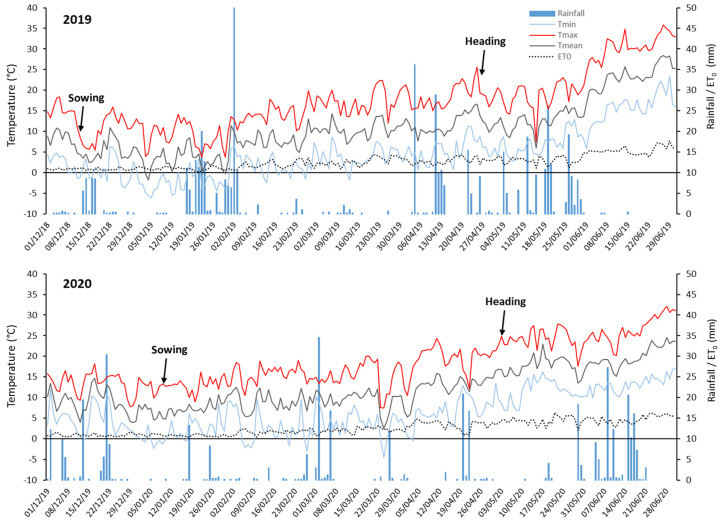
Meteorological data at the experimental site in Viterbo (Italy) during the 2019 and 2020 growing seasons; Tmin, Tmax and Tmean, minimum, maximum and mean daily temperature, respectively; ET_0_, daily evapotranspiration.

The effects of alien segments’ presence on spike fertility and overall plant production stability were analysed in two growing seasons, 2018–19 and 2019–20 (hereafter referred to as 2019 and 2020, respectively), at the experimental farm of the University of Tuscia, located in Viterbo, Italy (42°25′ N, 12°4′ E). Meteorological data during the growing seasons were retrieved from the meteorological station at the experimental site (Figure 3). Plants were sown on 11 December 2018, and 9 January 2020, in the two seasons, respectively, and grown under rainfed conditions in 2019, while rain-fed and irrigated conditions were compared in 2020. The irrigation treatment in 2020 consisted of 4 applications, starting from the onset of anthesis (i.e., on May 5, 12, 22 and 25). For each application, the amount of water necessary to replace the total evapotranspiration (ET) net of precipitations was applied. Total ET was calculated by adding up daily ET occurred between two subsequent irrigations (Figure 3), according to FAO-56 single crop coefficient procedures [45]. Common agronomic practices (fertilization, weed control) for durum wheat cultivation were adopted in all trials (see, e.g., [28]). Natural disease pressure was negligible in both experimental years; therefore, no fungicide treatment was applied.

Single plants (sp, Table 1) were arranged in triplicated rows (13 plants per row), at 25 cm distance between the rows and 10 cm between plants along the row. Five to eight plants per row were sampled for measurements of whole plant traits at maturity: tiller number (TILN), grain number (GN), grain yield (GY) per plant and 1000-grain weight (TGW). Three spikes per each plant were used for measurements of fertility parameters: spikelet No./spike (SPN), grain No./spike (GNS), grain No./spikelet (GNSP) and grain yield/spike (GYS). In the case of the 7el_1_L + 7EL recombinants and their controls grown in 2019 (Table 1), five spike-rows (sr) were arranged at 25 cm distance and five to 10 spikes sampled from each row for fertility analysis only. In 2020, an additional set of physiological traits was recorded under the rainfed and irrigated conditions: Chlorophyll content at anthesis (SPAD1), at 10 (SPAD2) and 20 (SPAD3) days post-anthesis (dpa) by using a hand-held meter SPAD 502 (Konica-Minolta Inc., Hino-shi Tokyo, Japan); quantum yield of photosystem II (PSII) of light-adapted flag leaves at 10 dpa (QY2) and 20 dpa (QY3), and photosynthetically active radiation (PAR), at anthesis (PAR1) and 20 dpa (PAR3), both measured by a portable fluorometer PAR-Fluorpen 110-LM/D (Photon Systems Instruments, Drasov, Czech Republic).

### 2.3. Statistical Analysis

One-way analysis of variance (ANOVA) was used to estimate differences between the genotypes (G) under rainfed conditions, while two-factor ANOVA was applied when G effect was analysed together with year (Y) or irrigation treatment (I) effects. In the 2020 analysis of the irrigation effect, replica (R) was included as a co-variant. Whenever a significant F value was obtained for single factors or their interactions, the Tukey HSD test was performed at 0.05 level. Normality of data was assessed for each variable (trait analysed) by the Kolmogorov-Smirnov test. In the case of test significance, data were log-transformed before ANOVA. All analyses were performed by SYSTAT12 software (Systat Software Inc., San Jose, CA, USA).

## 3. Results and Discussion

### 3.1. 7el_2_ Effect on Yield-Related Traits under Rainfed Conditions

The two-year analysis did not reveal any yield penalty of R193+ and R216+ RLs vs. their hom− controls. This indicates that the composite 7el_1_L + 7el_2_L alien segments on 7AL arms are well tolerated and do not cause negative effects on the fitness of the recipient tetraploid genotypes. Genotype effect (G), i.e., the presence/absence of a given alien segment, was more evident for spike than for plant traits (Table 2), the latter being under the influence of more numerous and interacting genetic and environmental factors. Independently from the alien segment presence, a positive background effect was noticed for grain yield/plant (GY) in R193 vs. R216 lines (about 20% increase, Table 2). Since RLs analysed here were selected in selfed progenies after a single cross to IDYT22 of BC_5_ RLs in Simeto background, a variable effect on phenotype due to random background allele assortments was expected. This might have masked R112-specific advantages detected at the same Viterbo growing site in previous trials involving the original R112+ vs. R5+ recombinants, which had highlighted higher tiller number, flag leaf area and bigger root apparatus in the former genotype [37,38,40]. From the traits analysed here, R193+ maintained a higher tiller number (TILN) with respect to R193- plants (albeit the difference was not significant), while SPN and GNS were somewhat reduced (−6% and −12%, respectively). Overall, the trade-off among yield-contributing traits of alien and background derivation led to equivalent grain yield/spike (GYS) and grain yield/plant (GY) in both R193+ vs. R193- and R216+ vs. R216- comparisons (Table 2).

**Figure 4:**
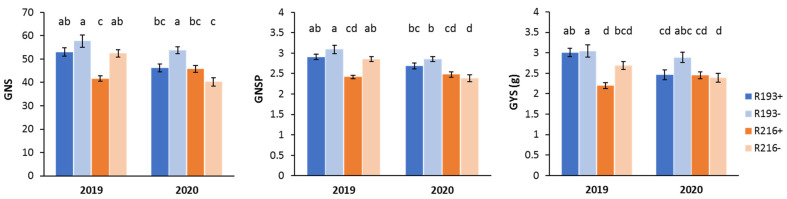
Significant Genotype × Year effect on spike yield traits measured in 2019 and 2020 for 7el_1_ + 7el_2_ recombinants (hom+) and their controls (hom−) (GNS, grain No./spike; GNSP, grain No./spikelet; GYS, grain yield/spike).; error bars represent standard errors of means; letters above the histograms correspond to the ranking of the Tukey test at *p* < 0.05 level.

When the year (Y) effect was considered, this turned out to be significant for virtually all traits of 7el_1_L + 7el_2_L RLs (Table 2). Remarkably different meteorological conditions characterized the 2019 and 2020 seasons, particularly in terms of precipitations (Figure 3). Season 2020 was much drier, with about three times less rainfall during the growing season with respect to 2019 (178 mm versus 486 mm, respectively). Moreover, during May 2019 an unusually high amount of rainfall and lower temperatures were recorded in coincidence with the critical phases of anthesis and grain filling. These conditions are probably at the basis of the higher values observed for all traits in the 2019 season, first and foremost for grain and tiller number, rather than grain weight (Table S1). However, the between-year difference highlighted an overall differential performance of the R193 and R216 types to the contrasting environmental conditions (Table S1). This was particularly evident for spike traits, showing a highly significant G × Y interaction (Table 2). For such traits, the R193 type exhibited similar values across the seasons (Figure 4), which is indicative of good yield stability. Only for the grain number/spike (GNS) trait, the presence of the R193 segment showed some depressing effect in the drier 2020 year. This is in line with previous results [37,38], which suggested the original R112 segment (retained in R193, see Figure 1) to carry in its most proximal 7el_1_L fraction genes/QTL for morpho-physiological and agronomical attributes that are best expressed in environments with optimal thermo-pluviometric patterns. On the other hand, the R216+ recombinant (R5 derivative) showed to be disadvantaged versus its R216- control in the cooler and rainier 2019 for grain number/spike (GNS), grain number/spikelet (GNSP) and grain yield/spike (GYS) (15% to 20% reduction; Figure 4), which indicates more investment by the plants in biomass than in grains. By contrast, R216 hom+ plants had comparable or higher values for these and other traits vs. R216 hom− plants in the hotter and drier 2020 season, when the allocation of assimilates was expected to be more sink- than source-driven. Since R216+ was developed from the R5+ line, previously shown to have better yield and biomass in some hot and dry environments [38], the same adaptive effect of the 7el_1_L chromatin might be maintained in both recombinant types. Moreover, the superior allocation of assimilates in sink versus source organs under unfavourable conditions is one of the main strategies to increase fruiting efficiency and yield [46].

### 3.2. 7E Effect on Yield-Related Traits under Rainfed Condition

Due to more recent introgression into durum wheat of the *Th. elongatum Fhb7E* QTL [33] with respect to the *Th. ponticum Fhb7el_2_* QTL [32], hence with limited seed availability for all the four 7el_1_L + 7EL recombinant types isolated, extensive field evaluation of their productivity was not possible so far. The first results on spike fertility traits of two R112-derived recombinant types (R69-9/R112 and R74-10/R112), grown in the 2018 and 2019 seasons, revealed an overall stable performance and no yield penalties due to the alien segment presence [33]. Here, a wider 2019 dataset was considered, including the two mentioned recombinants and a third type, named R74-10/R5, along with their hom− controls, all grown in spike rows (Table 1). ANOVA showed that differences due to the genotype were significant for all traits (Table 3). In general, the presence of any of the three alien segments did not cause negative outcomes on spike fertility or yield in 2019, as shown from the Tukey test of each hom+ vs. hom− pair. Positive background effect, as indicated by higher absolute values, on grain yield/spike (GYS), spikelet number/spike (SPN), grain number/spike (GNS) and grain number/spikelet (GNSP), were observed for R69-9/R112 (+/−) compared with the other two pairs. Moreover, in R74-10/R112+ and R69-9/R112+, the presence of their 7el_1_L + 7EL segments significantly increased GNS, GNSP and, consequently GY (+11% to +19%), when compared with the hom− controls (Table 3). Out of the three recombinants, R69-9/R112+ showed the highest spike yield (GYS) and overall yield potential, primarily determined by higher grain number, while R74-10/R5+ seemed to be the least productive. The latter may be associated with the bigger 7EL/7el_1_L chromatin ratio in R74-10/R5+ than in R74-10/R112+, with the larger 7EL segment of R74-10 derivatives possibly causing more disturbance to the recipient genome in comparison to that of R69-9 derivatives (Figure 1; [34]). The latter types thus appear to be more appropriate for durum wheat breeding, not least because in them the shorter 7EL chromosome stretch does not replace the 7el_1_ allele of the *Yp* gene, responsible for a considerable increase in semolina yellowness [28,47] vs. the corresponding and “paler” 7E allele [34].

Based on the 2019 results, in the following year the two R69-9-derived recombinants (R69-9/R112 and R69-9/R5), represented by BC_2_F_4_ progeny meanwhile obtained, were assayed. Consistent with the 2019 season, the 2020 trial did not show significant yield penalties of the 7el_1_L + 7EL hom+ lines (see GY, GYS, Table 3), indicating good production stability also under drier and warmer growing conditions. Although the differences for plant grain yield (GY) were not significant, the R69-9/R5+ recombinant line overcame its hom− control and R69-9/R112+ by 56% and 19%, respectively. This was likely due to the simultaneous increases of major GY components, such as TILN, GN and TGW (+18% for the latter, significant difference). Interestingly, grain weight increase was similarly observed in association with the 7el_1_L introgression of the original R5+ recombinant [47]. On the other hand, under the environmental conditions of 2020, R69-9/R112+ did not confirm the significant increase in grain number/spike expressed in 2019. However, this was not at the expenses of grain yield/spike, which showed high stability across the two years (Table 3).

As a whole and similarly to 7el_1_L + 7el_2_L segments (see Section 3.1), introgression of 7el_1_L + 7EL segments appears to be well tolerated in durum wheat and even increase yield, although effects on yield-related traits specifically ascribable to 7el_2_L- or 7EL-segment presence were not identified. The observed increases in yield-related traits were likely the result of positive interactions between the “nested” wild introgressions, as well as between them and the durum wheat background. For example, across the two years, higher grain number/spike (GNS) was consistently displayed by R112-derived 7el_1_L + 7EL RLs vs. other hom+ RLs, which may lead to the hypothesis that genetic factors for grain number are present in the 7el_1_ chromatin unique to R112+ (23–28% portion). Yet, such an effect was never observed in the primary R112+ recombinant across multiple and contrasting environments [29,37,38], suggesting a possible positive interaction of the 23–28% 7el_1_ chromatin of R112+ with the nested 7EL one, and/or with alleles in the durum background. To confirm this, however, further multi-environment and larger-scale trials will be needed.

### 3.3. Novel Alien Segment Effects on Physiological Traits

**Figure 5:**
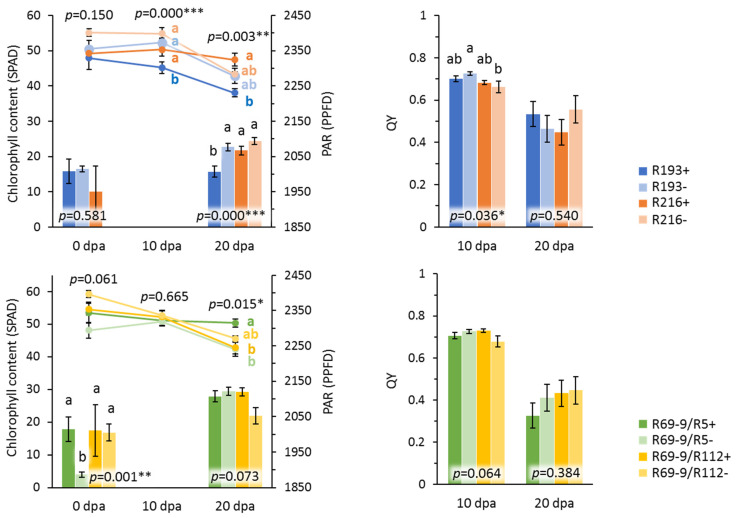
Flag leaf physiological traits measured for 7el_1_L + 7el_2_L and 7el_1_L + 7EL recombinants (hom+) and their controls (hom−) in 2020 (PAR, photosynthetically active radiation; PPFD, photosynthetic photon flux density; QY, quantum yield of PSII). On the left, line graphs represent chlorophyll content, and histograms represent PAR; error bars represent standard errors of means; *, **, ***, significance at *p* < 0.05, 0.01 and 0.001 level, respectively.

Light interception and radiation use efficiency are major source-related components of wheat yield [48]. Thus far, most breeding improvements of wheat yield have been obtained by increasing yield sink, i.e., grain yield (number and weight), while breeding for yield source has still room for improvement [49,50]. Optimisation of the source-sink relations through the increase of light interception capacity and radiation use efficiency is considered one of the main strategies in breeding for yield improvement [48,51,52,53]. Based on these considerations, in 2020 we have assessed a set of additional physiological parameters related to photosynthesis of flag leaves on all hom+ recombinant and control genotypes, to possibly identify new *Thinopyrum* loci involved in their control. With respect to the yield-related traits described above, flag leaf chlorophyll content (SPAD) and in particular light interception (PAR) and PSII efficiency (QY), were not consistently associated with the presence/absence of alien introgressions (Figure 5). Only in the cases of R193+ and R69-9/R5+ the presence of their alien segments significantly affected SPAD and PAR when compared to their hom− controls, particularly at advanced grain filling stages. R193+ had significantly lower SPAD at 10 dpa (−14%) and PAR at 20 dpa (−3%), while R69-9/R5+ had increased SPAD at 20 dpa (+19%) and PAR at anthesis (+7%). The latter is indicative of the existence of putative genetic factor(s) within the 7EL segment in R69-9/R5+ that improve(s) light interception, i.e., source strength in the pre-anthesis period. This represents a limiting factor in wheat, which is crucial for the accumulation of biomass to be relocated into spikes and grains ([54] and reference therein).

Previous studies on the original R5 and R112 recombinants [37,38] identified the presence of QTL for an increase of SPAD, stay-green trait and flag leaf dimensions in the proximal 23–28% fraction of 7el_1_L specific to R112 (see Figure 1). An increase of SPAD was similarly detected in the R69-9/R112+ derived recombinant, as opposed to a decrease in R193+ vs. their respective controls (Figure 5). This indicates no detrimental effect of the 7EL chromosome portion nested in the former recombinant, which is in contrast with a depressing effect of the 7el_2_L portion present in the latter. Since photosynthesis-related traits are under strong environmental effect [54], the hypothesized 7el_1_/7el_2_/7E roles will be further assessed.

### 3.4. Yield Performance of Durum Wheat-Thinopyrum spp. Recombinants vs. Top-Yielding Cultivars under Rainfed and Irrigated Conditions

Identifying putative QTL for yield traits within alien introgressions is of great importance for the development of functional molecular markers for selection and genetic studies. However, to evaluate the recombinants’ real yielding potential is essential to build up an efficient breeding pipeline. The expression of any gene for quantitative traits, e.g., yield-related, is dependent on a complex system of interactions between plant genome, physiology and environment. Therefore, new genotypes should be evaluated in comparison with good yielding and adapted varieties. There is no certainty that a positive effect of an alien gene/QTL observed in a hom+ vs. hom− comparison will result in superior final yield when compared with well-adapted cultivated wheats. Therefore, in 2020, we tested at small-scale the best four of the secondary recombinants with pyramided *Thinopyrum* spp. segments against a set of known high-yielding varieties, well-adapted to durum wheat cultivation areas similar to our experimental site [42]. Sixteen yield-related traits were recorded under rainfed and irrigated growing condition (Table 4 and Table 5) and yield potential evaluated under the genotype × treatment interaction.

Grain yield/plant (GY) of all secondary hom+ recombinants (RLs) did not show to be statistically different from that of other genotypes, thus confirming the absence of negative effects by alien introgressions (Table 4). Moreover, all recombinants showed 9–47% higher GY when compared to their founder genotypes i.e., R5+, R112+, Simeto and IDYT22, and 3 of them were more productive than Monastir, one of the best yielders in Italy [42]. Based on the results of specific yield-contributing traits, increases of GY were associated with increases in plant sink traits, such as tiller and grain number (TILN, GN), as well as grain number/spike (GNS). For these traits, all secondary RLs showed superior values to the trait means, resulting significantly higher than those of at least one founder genotype (Table 4). In contrast, 1000-grain weight had differential and minor importance for GY increase. The most productive recombinants were the R5-derived R216+ and R69-9/R5+, followed by R69-9/R112+ and the slightly less productive R193+. The results undoubtedly indicate that the presence of composite alien segments enhanced the yield potential of the RLs with respect to that of R5+ and R112+ primary recombinants, carrying 7el_1_L chromatin only, and of durum wheat cultivars. This further sustains the hypothesis of positive interactions between the introgressed segments from different *Thinopyrum* spp. donors and the recipient genome (see above, Section 3.2). For example, a significant positive influence of the nested segments was revealed for some spike sink traits in comparison with the respective primary 7el_1_L segment. Grain number/spike (GNS) showed to be significantly higher (+15%) in R216+ (7el_1_L + 7el_2_L) and in R69-9/R112+ (7el_1_L + 7EL) than in their primary types (R5+ and R112+, respectively). The same 7EL segment also significantly increased grain yield/spike (GYS) in R69-9/R112+ vs. R112+ (+16%). Finally, spike fertility (SFI) was boosted by 9% in R69-6/R5+ when compared to R5+, due to the 7EL segment presence (Table 4).

Interestingly, the best yielding RLs in 2020, R216+ and R69-9/R5+, showed to have their yields (GY, GYS) determined by different key components, i.e., they adopt different physiological mechanisms in forming final yield. In the case of R216+, yield formation is apparently achieved by harnessing the grain number/spike (GNS) and spike fertility (GNSP, SFI) traits, whereas R69-9/R5+ exploits the seed weight (TGW) path. Notwithstanding this, by taking into account values and significances for all measured traits in Table 4, it was the R69-9/R112+ recombinant (Figure 2) to exhibit the best productive potential when compared to cultivated varieties. Besides the fact that this was the only RL with all trait values superior to the trial means, both source and sink traits were enhanced in the presence of its 7el_1_L + 7EL *Thinopyrum* introgression, particularly the spike sink traits GNS and GYS, but also GNSP and SFI. As mentioned in Section 3.2, a higher grain number/spike was never observed in the original R112+ RL, which suggests GNS in R69-9/R112+ to be correlated with the presence of the 7EL chromosome portion. Moreover, the ability of the hom+ line to maintain its GNS value comparable to that of its hom− in the less favourable 2020 season (Table 3), and to result significantly better than the founder genotypes (see Table 1) and other cultivars, such as Kanakis (Table 4), is indicative of its good adaptation to conditions with reduced water availability. Increasing grain number in a dry and hot environment represents a positive adaptive mechanism of remobilization of assimilates from source organs (tillers, leaves) into grains [46]. The primary R112+ recombinant is known to have a larger photosynthetically active leaf area and higher chlorophyll content at advanced grain filling stage [29,37,38], as well as a deeper and wider root apparatus with respect to other durum wheat-*Th. ponticum* 7el_1_ recombinants [40]. This is in line here with the present results of flag leaf traits (Table 5), which confirm chlorophyll content and correlated PAR to be superior at 20 dpa in R112+ and its derivative R69-9/R112+ with respect to other genotypes. This was particularly evident under rainfed conditions vs. irrigated conditions, as shown by the Tukey test of significant G × I interaction (Figure S1). All flag leaf physiological traits showed less variability due to the alien segment presence, the four secondary recombinants being essentially similar between each other and to durum cultivars (Table 5).

Supporting evidence to the good adaptability of the R69-9/R112+ genotype also comes from the results of the analysis of irrigation treatment effect (Figures S1 and S2). In general, irrigation (I) alone or in combination with genotype (G × I) was highly significant for most of the traits (Table 4 and Table 5). However, the Tukey test revealed relevant differences only in few cases, for grain number (GN, GNS) and yield (GY, GYS) (Figure S2), which are essential traits for yield determination under contrasting meteorological conditions. Although higher values were recorded for all traits in the irrigated vs. rain-fed condition, for most genotypes included in our experiment these variations were minimal and suggested their yield traits to be more dependent on genotypic attributes rather than on the tested environmental factors. Nonetheless, R69-9/R112+ was among the few lines displaying different phenotypes under the two growing conditions. For instance, its largest advantageous effect under the less favourable rain-fed conditions was confirmed for GYS, which was 24–40% superior in R69-9/R112+ than in 8 out of the other 11 genotypes (all except Antalis, R5+ and R69-9/R5+; Figure S2, Tukey test). As tiller number was unaffected by the G × I interaction (Table 4), the higher GYS under drought-prone conditions likely allowed R69-9/R112+ recombinant to maintain total plant yield (GY) at high levels, possibly through more efficient assimilate transfer to grains. *Thinopyrum* species are adapted and even cultivated in harsh and dry environments [55], and numerous genes/QTL that improve adaptation to environmental stresses, including drought, have been identified in the species themselves (e.g., [56]) and in their introgression products into wheat (e.g., [57,58,59,60]). Nevertheless, to confirm the present results and to describe the nature of the suggested better adaptability, further research and field trials across diverse environments are underway.

## 4. Conclusions and Perspectives

The small-sized, composite alien segments, in which portions of homoeologous group 7 chromosomes from different species or accessions of the *Thinopyrum* genepool were nested, proved not to upset yield performance of recipient durum wheat, and even to have the potential to improve it under both favourable and stressful environmental conditions. Based on the observed results, there may well be a complex network of interactions between environmentally modulated “wild” and “cultivated” factors of both genetic and epigenetic nature. Such factors were similarly contemplated to explain the novel phenotypes exhibited by cultivated germplasm, containing alien/wild introgressions (e.g., [28,61]). In addition to these direct effects on yield, an indirect contributing asset is provided to the novel RLs by the introduced genes/QTL for resistance against leaf rust (*Lr19*) as well as Fusarium threats, i.e., FHB and FCR (*Fhb7el*_2_ and *Fhb7E*). Notably, among wheat diseases, leaf rust and FHB are ranked first and second in terms of yield losses globally determined [62]. The presence of the *Yp* gene, increasing yellow pigmentation of semolina and pasta products, whose most effective *Th. ponticum* allele is present in all advanced RL selections (Table 1 and Table 5), adds value to their end-use quality. As a whole, the novel RLs described here, prove to have excellent characteristics for exploitation in sustainable breeding and cultivar development addressed to different environments. To speed up this process, the use of friendly molecular markers for alien segment selection (see, e.g., [32,33]), coupled with the rapid generation advancement system ‘speed breeding’, e.g., [63], can be profitably deployed.

## Figures and Tables

**Table 1:** Durum wheat-*Thinopyrum* spp. recombinants analysed in the present study (RF, rainfed; IRR, irrigated; sp, spaced plants; sr, spike-row).

Recombinant	Pedigree	Progeny	Alien Segment		Alien Genes				Field Trial	
			Presence	%7AL	*Lr19*	*Yp*	*Fhb-7el2*	*Fhb-7EL*	RF	RF + IRR
R112+	(Tr#12 ^a^ × Creso *ph1c* ^b^)/5*Simeto	BC_5_F_11_	hom+	28	+	+	-	-		2020 (sp)
R5+	(Tr#12 × Creso *ph1c*)/5*Simeto	BC_5_F_11_	hom+	23	+	+	-	-		2020 (sp)
R193+	(KS24-1 ^c^ × R112+)/Simeto/IDYT22	F_4-5_	hom+	28	+	+	+	-	2019 (sp)	2020 (sp)
R193-	(KS24-1 × R112+)Simeto/IDYT22	F_4-5_	hom−	-	-	-	-	-	2019 (sp)	2020 (sp)
R216+	(KS24-1 × R5+)/Simeto/IDYT22	F_4-5_	hom+	23	+	+	+	-	2019 (sp)	2020 (sp)
R216-	(KS24-1 × R5+)/Simeto/IDYT22	F_4-5_	hom−	-	-	-	-	-	2019 (sp)	2020 (sp)
R74-10/R112+	(R74-10 ^d^ × R112+)/2*Simeto	BC_2_F_4_	hom+	28	+	-	-	+	2019 (sr)	
R74-10/R112-	(R74-10 × R112+)/2*Simeto	BC_2_F_4_	hom−	-	-	-	-	-	2019 (sr)	
R74-10/R5+	(R74-10 × R5+)/2*Simeto	BC_2_F_4_	hom+	23	+	-	-	+	2019 (sr)	
R74-10/R5-	(R74-10 × R5+)/2*Simeto	BC_2_F_4_	hom−	-	-	-	-	-	2019 (sr)	
R69-9/R112+	(R69-9 ^d^ × R112+)/2*Simeto	BC_2_F_4-5_	hom+	28	+	+	-	+	2019 (sr)	2020 (sp)
R69-9/R112-	(R69-9 × R112+)/2*Simeto	BC_2_F_4-5_	hom−	-	-	-	-	-	2019 (sr)	2020 (sp)
R69-9/R5+	(R69-9 × R5+)/2*Simeto	BC_2_F_4_	hom+	23	+	+	-	+		2020 (sp)
R69-9/R5-	(R69-9 × R5+)/2*Simeto	BC_2_F_4_	hom−	-	-	-	-	-		2020 (sp)

^a^ Bread wheat translocation line with 7AL and 61% proximal 7AS arms replaced by *Th. ponticum* 7el_1_ chromatin [36]. ^b^ Mutant line of durum wheat cv. Creso for the *Ph1* gene on the 5BL arm that controls homoeologous pairing [44]. ^c^ Bread wheat translocation *Fhb7el*_2_ donor line with the 7DL arm replaced by *Th. ponticum* 7el_2_ chromatin [32]. ^d^ Bread wheat recombinant lines with 70% of 7DL arm replaced by an alien segment composed of *Th. ponticum* 7el_1_L and *Th. elongatum* 7EL chromatin [34]. Both lines harbour the *Fhb7E* QTL.

**Table 2:** Mean values and ANOVA of yield and yield-related traits recorded for 7el_1_L + 7el_2_L recombinants (hom+) and their controls (hom−) across the 2019 and 2020 seasons.

Genotype	Plant Traits				Spike Traits			
	GY (g)	TILN	GN	TGW (g)	GYS (g)	SPN	GNS	GNSP
R193+	13.3	7.5	265.5	50.2	2.7 ab	17.5 b	48.9 b	2.8 ab
R193-	13.3	6.9	278.0	49.8	2.9 a	18.7 a	55.3 a	2.9 a
R216+	11.1	7.0	226.3	50.3	2.4 c	18.0 b	44.2 c	2.5 c
R216-	11.1	7.4	223.1	52.6	2.5 bc	17.3 b	44.8 bc	2.6 bc
ANOVA *p*								
Genotype (G)	0.090	0.980	0.040 *	0.681	0.000 ***	0.001 **	0.000 ***	0.000 ***
Year (Y)	0.000 ***	0.000 ***	0.000 ***	0.000 ***	0.025 *	0.280	0.000 ***	0.000 ***
G × Y	0.147	0.241	0.107	0.040 *	0.007 **	0.000 ***	0.000 ***	0.007 **

GY, grain yield/plant; TILN, tiller number/plant; GN, grain number/plant; TGW, 1000-grain weight; GYS, grain yield/spike; SPN, spikelet No./spike; GNS, grain No./spike; GNSP, grain No./spikelet; letters in each column correspond to ranking of Tukey test at 0.05 level for genotype effect; *, ** and ***, significance at *p* < 0.05, 0.01, and 0.001.

**Table 3:** Mean values and ANOVA of yield and yield-related traits recorded for 7el_1_L + 7EL recombinants (hom+) and their controls (hom−) in the 2019 and 2020 seasons.

Genotype	Plant Traits				Spike Traits				
	GY (g)	TILN	GN	TGW (g)	GYS (g)	SPN	GNS	GNSP	SFI
2019 (sr)									
R69-9/R112+	-	-	-	49.6 ab	3.0 a	19.2 ab	61.2 a	3.2 a	55.3 a
R69-9/R112-	-	-	-	51.8 a	2.8 ab	20.0 a	54.9 b	2.7 bc	52.7 ab
R74-10/R112+	-	-	-	53.9 a	2.7 b	17.9 cd	49.5 bc	2.8 b	46.5 c
R74-10/R112-	-	-	-	52.2 a	2.3 c	17.3 d	44.2 d	2.5 cd	50.7 ac
R74-10/R5+	-	-	-	45.1 b	2.1 c	18.8 bc	47.3 cd	2.5 d	51.8 ab
R74-10/R5-				47.8 ab	2.2 c	18.3 bd	47.3 cd	2.6 bd	49.7 bc
ANOVA *p*	-	-	-	0.000 ***	0.000 ***	0.000 ***	0.000 ***	0.000 ***	0.001 **
2020 (sp)									
R69-9/R112+	11.3	4.2	191.2	59.1 ab	3.0 ab	18.4 b	50.7 ab	2.7 a	58.7 b
R69-9/R112-	11.2	4.0	199.6	56.6 bc	3.3 a	20.1 a	55.9 a	2.8 a	68.1 a
R69-9/R5+	13.4	5.0	213.5	63.4 a	2.9 bc	19.6 a	45.6 b	2.3 b	54.7 b
R69-9/R5-	8.6	4.0	159.9	53.7 c	2.6 c	19.7 a	46.7 b	2.4 b	64.0 a
ANOVA *p*	0.178	0.325	0.560	0.000 ***	0.000 ***	0.000 ***	0.000 ***	0.000 ***	0.000 ***

GY, grain yield/plant; TILN, tiller number/plant; GN, grain number/plant; TGW, 1000-grain weight; GYS, grain yield/spike; SPN, spikelet No./spike; GNS, grain No./spike; GNSP, grain No./spikelet; SFI, spike fertility index.; sr, spike row; sp, spaced plants; letters in each column correspond to the ranking of Tukey test at 0.05 level for Genotype effect; ** and ***, significance at *p* < 0.01 and 0.001, respectively.

**Table 4:** Mean values and ANOVA of yield and yield-related traits recorded in 2020 for 7el_1_L + 7el_2_L and 7el_1_L + 7EL durum wheat RLs and control cultivars. Genotypes are ordered from the best to the least productive one according to GY.

Genotype	Plant Traits				Spike Traits				
	GY (g)	TILN	GN	TGW (g)	GYS (g)	SPN	GNS	GNSP	SFI
R216+	**13.5**	**5.9 a**	**239.4 a**	56.4 de	**2.8 bd**	**18.3 ce**	**48.9 ab**	**2.7 ad**	**62.0 a**
R69-9/R5+	**13.4**	**5.0 ac**	**208.2 ac**	**64.8 ab**	**3.0 ab**	**19.1 bc**	45.9 bd	2.4 e	54.0 ab
R69-9/R112+	**13.3**	**4.8 ac**	**222.0 ab**	**59.8 bd**	**3.2 a**	**18.4 cd**	**52.6 a**	**2.9 a**	**61.8 a**
Monastir	**12.6**	**5.2 ac**	**231.7 ab**	54.3 e	2.7 be	**19.6 b**	**49.3 ab**	2.5 ce	**59.1 ab**
R193+	**12.0**	**5.0 ab**	**206.2 ac**	58.3 de	2.7 be	17.1 f	**46.1 bd**	**2.7 ac**	52.4 ab
Ettore	**11.6**	**5.3 ab**	**222.3 ab**	52.5 e	2.5 e	**18.7 c**	**47.8 ac**	2.6 be	**59.8 e**
R5+	11.0	4.5 ac	172.1 ac	**63.1 ac**	2.7 be	16.0 g	42.6 de	**2.6 ad**	49.5 bc
R112+	10.7	4.4 bc	185.9 ac	58.3 ce	2.7 ce	15.8 g	45.2 bd	**2.8 ab**	**61.8 a**
Kanakis	10.6	4.7 ac	185.5 ac	56.3 de	2.5 de	17.4 ef	43.3 cd	2.5 ce	54.9 ab
IDYT22	10.2	4.6 ac	157.8 bc	**64.3 ab**	2.5 e	16.1 g	37.9 e	2.4 e	42.0 c
Antalis	9.8	4.0 bc	180.3 ac	55.5 de	**2.9 ac**	**20.6 a**	**50.2 ab**	2.4 e	**58.4 ab**
Simeto	9.2	3.3 c	134.8 c	**66.7 a**	**2.8 ac**	17.5 df	41.4 ce	2.4 de	51.4 ac
Mean	11.5	4.7	195.5	59.2	2.7	17.9	45.9	2.6	55.6
ANCOVA *p*									
Genotype (G)	0.031 *	0.000 ***	0.000 ***	0.000 ***	0.000 ***	0.000 ***	0.000 ***	0.000 ***	0.000 ***
Irrigation (I)	0.000 ***	0.000 ***	0.000 ***	0.000 ***	0.000 ***	0.322	0.000 ***	0.000 ***	0.717
G × I	0.019 *	0.078	0.019 *	0.014 *	0.000 ***	0.005 **	0.000 ***	0.000 ***	0.174
Replica	0.000 ***	0.109	0.002 **	0.015 *	0.000 ***	0.000 ***	0.000 ***	0.016 *	0.695

GY, grain yield/plant; TILN, tiller number/plant; GN, grain number/plant; TGW, 1000-grain weight; GYS, grain yield/spike; SPN, spikelet No./spike; GNS, grain No./spike; GNSP, grain No./spikelet; SFI, spike fertility index; letters in each column correspond to the ranking of Tukey test at *p* < 0.05 level; *, ** and ***, significance at *p* < 0.05, 0.01 and 0.001 level, respectively; in bold, values above the trait mean.

**Table 5:** Mean values and ANOVA of flag leaf traits recorded in 2020 for 7el_1_L + 7el_2_L and 7el_1_L + 7EL durum wheat RLs and control cultivars. Genotypes are ordered from the best to least productive according to GY in Table 4.

Genotype	Flag Leaf Traits						
	SPAD1	SPAD2	SPAD3	PAR1	PAR3	QY2	QY3
R216+	49.2 ab	50.7 ab	**50.9 ab**	**1950.0**	**2055.0 ac**	**0.69 ac**	0.46 ab
R69-9/R5+	**53.5 ab**	**51.8 ab**	**52.1 a**	**2014.4**	**2098.3 a**	**0.71 a**	0.42 ab
R69-9/R112+	**54.5 ab**	**52.7 ab**	46.3 ad	**2011.1**	**2093.3 ab**	**0.71 a**	0.46 ab
Monastir	49.6 ab	50.3 ab	46.1 ad	1845.6	2011.7 cf	**0.72 a**	**0.51 ab**
R193+	47.9 ab	48.8 b	44.1 cd	**2007.8**	**2023.3 ce**	**0.70 ab**	**0.54 ab**
Ettore	45.7 b	50.5 ab	44.5 bd	1796.7	2016.7 cf	0.66 bc	0.47 ab
R5+	**52.0 ab**	**51.6 ab**	**48.1 ad**	**2025.6**	1975.8 df	0.68 ac	0.45 ab
R112+	51.5 ab	**52.7 ab**	**52.4 a**	1893.3	**2103.0 ab**	**0.70 ab**	0.46 ab
Kanakis	45.5 b	49.3 ab	41.9 d	1841.1	1912.5 g	**0.69 ac**	0.44 ab
IDYT22	**56.7 ab**	**51.6 ab**	44.9 bd	**2020.0**	1951.7 fg	**0.69 ac**	**0.49 ab**
Antalis	**52.9 ab**	**52.1 ab**	42.2 cd	N/A	**2031.7 bd**	0.67 ac	**0.59 a**
Simeto	**60.6 a**	**54.3 a**	**48.9 ac**	1923.3	1971.1 ef	0.64 c	0.41 b
Mean	51.6	51.4	46.9	1939.0	2020.3	0.69	0.47
ANCOVA *p*							
Genotype (G)	0.006 **	0.041 *	0.000 ***	0.055	0.000 ***	0.000 ***	0.038 *
Irrigation (I)	-	0.008 **	0.000 ***	-	0.063	0.245	0.000 ***
G × I	-	0.629	0.009 **	-	0.000 ***	0.065	0.360
Replica	0.647	0.788	0.049 *	0.014 *	0.681	0.268	0.486

SPAD, chlorophyll content; PAR, photosynthetically active radiation; QY, quantum yield of PSII; 1, at anthesis; 2, at 10 days post-anthesis; 3, at 20 days post-anthesis; letters in each column correspond to the ranking of Tukey test at *p* < 0.05 level; *, ** and ***, significance at *p* < 0.05, 0.01 and 0.001 level, respectively; in bold, values above the trait mean; N/A, not available.

## Supplementary Materials

The following are available online at https://www.mdpi.com/2223-7747/10/3/579/s1, Figure S1: Significant Genotype × Treatment effect on flag leaf physiological traits measured in 2020 under rainfed and irrigated conditions, Figure S2: Significant Genotype × Treatment effect on yield-related traits measured in 2020 under rainfed and irrigated conditions, Table S1: Means ± standard errors of yield and yield-related traits recorded in 2019 and 2020 seasons for 7el1L + 7el2L recombinants (R193+, R216+) and their null segregates (R193-, R216-) under rainfed growing conditions.

## References

[1] Xynias I.N., Mylonas I., Korpetis E.G., Ninou E. (2020). Durum Wheat Breeding in the Mediterranean Region: Current Status and Future Prospects. Agronomy.

[2] FAOSTAT. http://www.fao.org/faostat/en/#data/QC.

[3] Sall A.T., Chiari T., Legesse W., Seid-Ahmed K., Ortiz R., van Ginkel M., Bassi F.M. (2019). Durum Wheat (*Triticum durum* Desf.): Origin, Cultivation and Potential Expansion in Sub-Saharan Africa. Agronomy.

[4] Martínez-Moreno F., Solís I., Noguero D., Blanco A., Özberk İ., Nsarellah N., Elias E., Mylonas I., Soriano J. (2020). Durum wheat in the Mediterranean rim: Historical evolution and genetic resources. Genet. Resour. Crop Evol..

[5] Maccaferri M., Harris N.S., Twardziok S.O., Pasam R.K., Gundlach H., Spannagl M., Ormanbekova D., Lux T., Prade V.M., Milner S.G. (2019). Durum wheat genome highlights past domestication signatures and future improvement targets. Nat. Genet..

[6] Tester M., Langridge P. (2010). Breeding technologies to increase crop production in a changing world. Science.

[7] Ray D.K., Ramankutty N., Mueller N.D., West P.C., Foley J.A. (2012). Recent patterns of crop yield growth and stagnation. Nat. Commun..

[8] Ray D.K., Mueller N.D., West P.C., Foley J.A. (2013). Yield Trends Are Insufficient to Double Global Crop Production by 2050. PLoS ONE.

[9] Senapati N., Brown H.E., Semenov M.A. (2019). Raising genetic yield potential in high productive countries: Designing wheat ideotypes under climate change. Agric. For. Meteorol..

[10] Xiong W., Reynolds M.P., Crossa J., Payne T.S., Schulthess U., Sonder K., Addimando N., Singh R.P., Ammar K., Gerard B. (2020). Climate change has increased genotype-environment interactions in wheat breeding. Res. Sq..

[11] Ceoloni C., Kuzmanović L., Forte P., Gennaro A., Bitti A. (2014). Targeted exploitation of gene pools of alien Triticeae species for sustainable and multi-faceted improvement of the durum wheat crop. Crop Pasture Sci..

[12] Gregory P.J., Johnson S.N., Newton A.C., Ingram J.S.I. (2009). Integrating pests and pathogens into the climate change/food security debate. J. Exp. Bot..

[13] Chakraborty S., Newton A.C. (2011). Climate change, plant diseases and food security: An overview. Plant Pathol..

[14] Zhang X., Halder J., White R.P., Hughes D.J., Ye Z., Wang C., Xu R., Gan B., Fitt B.D.L. (2014). Climate change increases risk of fusarium ear blight on wheat in central China. Ann. Appl. Biol..

[15] Alahmad S., Kang Y., Dinglasan E., Mazzucotelli E., Voss-Fels K.P., Able J.A., Christopher J., Bassi F.M., Hickey L.T. (2020). Adaptive traits to improve durum wheat yield in drought and crown rot environments. Int. J. Mol. Sci..

[16] Buerstmayr M., Steiner B., Buerstmayr H. (2020). Breeding for Fusarium head blight resistance in wheat—Progress and challenges. Plant Breed..

[17] Maresca M. (2013). From the gut to the brain: Journey and pathophysiological effects of the food-associated trichothecene mycotoxin deoxynivalenol. Toxins.

[18] Walkowiak S., Gao L., Monat C., Haberer G., Kassa M.T., Brinton J., Ramirez-Gonzalez R.H., Kolodziej M.C., Delorean E., Thambugala D. (2020). Multiple wheat genomes reveal global variation in modern breeding. Nature.

[19] Wang M., Wang S., Liang Z., Shi W., Gao C., Xia G. (2018). From Genetic Stock to Genome Editing: Gene Exploitation in Wheat. Trends Biotechnol..

[20] Brinton J., Ramirez-Gonzalez R.H., Simmonds J., Wingen L., Orford S., Griffiths S., Haberer G., Spannagl M., Walkowiak S., Pozniak C. (2020). A haplotype-led approach to increase the precision of wheat breeding. Commun. Biol..

[21] Ceoloni C., Kuzmanović L., Forte P., Virili M.E., Bitti A., Molnár-Láng M., Ceoloni C., Doležel J. (2015). Wheat-Perennial Triticeae Introgressions: Major Achievements and Prospects. Alien Introgression in Wheat—Cytogenetics, Molecular Biology, and Genomics.

[22] Feuillet C., Langridge P., Waugh R. (2008). Cereal breeding takes a walk on the wild side. Trends Genet..

[23] Mondal S., Rutkoski J.E., Velu G., Singh P.K., Crespo-Herrera L.A., Guzman C., Bhavani S., Lan C., He X., Singh R.P. (2016). Harnessing diversity in wheat to enhance grain yield, climate resilience, disease and insect pest resistance and nutrition through conventional and modern breeding approaches. Front. Plant Sci..

[24] Prohens J., Gramazio P., Plazas M., Dempewolf H., Kilian B., Díez M.J., Fita A., Herraiz F.J., Rodríguez-Burruezo A., Soler S. (2017). Introgressiomics: A new approach for using crop wild relatives in breeding for adaptation to climate change. Euphytica.

[25] Sears E.R., Kimber G., Rédei G.R. (1972). Chromosome engineering in wheat. Stadler Genetics Symposia.

[26] Sears E.R., Evans L.T., Peacock W.J. (1981). Transfer of alien genetic material to wheat. Wheat Science: Today and Tomorrow.

[27] Qi L., Friebe B., Zhang P., Gill B.S. (2007). Homoeologous recombination, chromosome engineering and crop improvement. Chromosom. Res..

[28] Kuzmanović L., Rossini F., Ruggeri R., Pagnotta M., Ceoloni C. (2020). Engineered Durum Wheat Germplasm with Multiple Alien Introgressions: Agronomic and Quality Performance. Agronomy.

[29] Kuzmanović L., Gennaro A., Benedettelli S., Dodd I.C., Quarrie S.A., Ceoloni C. (2014). Structural-functional dissection and characterization of yield-contributing traits originating from a group 7 chromosome of the wheatgrass species *Thinopyrum ponticum* after transfer into durum wheat. J. Exp. Bot..

[30] Sharma S., Xu S., Ehdaie B., Hoops A., Close T.J., Lukaszewski A.J., Waines J.G. (2011). Dissection of QTL effects for root traits using a chromosome arm-specific mapping population in bread wheat. Theor. Appl. Genet..

[31] Reynolds M., Langridge P. (2016). Physiological breeding. Curr. Opin. Plant Biol..

[32] Forte P., Virili M.E., Kuzmanović L., Moscetti I., Gennaro A., D’Ovidio R., Ceoloni C. (2014). A novel assembly of *Thinopyrum ponticum* genes into the durum wheat genome: Pyramiding Fusarium head blight resistance onto recombinant lines previously engineered for other beneficial traits from the same alien species. Mol. Breed..

[33] Kuzmanović L., Mandalà G., Tundo S., Ciorba R., Frangella M., Ruggeri R., Rossini F., Gevi F., Rinalducci S., Ceoloni C. (2019). Equipping Durum Wheat—*Thinopyrum ponticum* Recombinant Lines with a *Thinopyrum elongatum* Major QTL for Resistance to Fusarium Diseases Through a Cytogenetic Strategy. Front. Plant Sci..

[34] Ceoloni C., Forte P., Kuzmanović L., Tundo S., Moscetti I., De Vita P., Virili M.E., D’Ovidio R. (2017). Cytogenetic mapping of a major locus for resistance to Fusarium head blight and crown rot of wheat on *Thinopyrum elongatum* 7EL and its pyramiding with valuable genes from a *Th. ponticum* homoeologous arm onto bread wheat 7DL. Theor. Appl. Genet..

[35] Wang H., Sun S., Ge W., Zhao L., Hou B., Wang K., Lyu Z., Chen L., Xu S., Guo J. (2020). Horizontal gene transfer of Fhb7 from fungus underlies Fusarium head blight resistance in wheat. Science.

[36] Ceoloni C., Forte P., Gennaro A., Micali S., Carozza R., Bitti A. (2005). Recent developments in durum wheat chromosome engineering. Cytogenet. Genome Res..

[37] Kuzmanović L., Ruggeri R., Virili M.E., Rossini F., Ceoloni C. (2016). Effects of *Thinopyrum ponticum* chromosome segments transferred into durum wheat on yield components and related morpho-physiological traits in Mediterranean rain-fed conditions. Field Crop. Res..

[38] Kuzmanović L., Ruggeri R., Able J.A., Bassi F.M., Maccaferri M., Tuberosa R., De Vita P., Rossini F., Ceoloni C. (2018). Yield of chromosomally engineered durum wheat-*Thinopyrum ponticum* recombinant lines in a range of contrasting rain-fed environments. Field Crop. Res..

[39] Ceoloni C., Kuzmanović L., Ruggeri R., Rossini F., Forte P., Cuccurullo A., Bitti A. (2017). Harnessing genetic diversity of wild gene pools to enhance wheat crop production and sustainability: Challenges and opportunities. Diversity.

[40] Virili M., Kuzmanović L., Bitti A., Salvi S., Tuberosa R., Ceoloni C. Analysis of seminal root architecture in durum wheat-*Thinopyrum ponticum* recombinant lines. Proceedings of the Joint Meeting SIBV-SIGA.

[41] Rossini F., Provenzano M.E., Kuzmanović L., Ceoloni C., Ruggeri R. (2020). Assessing the Ability of Durum Wheat-*Thinopyrum ponticum* Recombinant Lines to Suppress Naturally Occurring Weeds under Different Sowing Densities. Agronomy.

[42] Quaranta F., Arcangeli A., Basili O., Belocchi A., Bottazzi P., Fabbrini L., Malagesi F., Mariotti R., Mazzon V., Cacciatori P. (2020). Speciale Grano Duro: Dettaglio regionale dei risultati 2020—Centro Italia versante tirrenico. L’informatore Agrar..

[43] Carboni G., Dettori M., Mameli L., Rinaldi M., Colecchia S.A., Belocchi A., Quaranta F. (2018). Prove di grano duro su sodo in Sardegna e Puglia. L’informatore Agrar..

[44] Giorgi B. Origin, behaviour and utilization of a *Ph1* mutant of durum wheat, *Triticum turgidum* (L.) var. *durum*. Proceedings of the 6th International Wheat Genetics Symposium.

[45] Allen R.G., Pereira L.S., Raes D., Smith M. (1998). Crop Evapotranspiration—Guidelines for Computing Crop Water Requirements.

[46] Slafer G.A., Elia M., Savin R., García G.A., Terrile I.I., Ferrante A., Miralles D.J., González F.G. (2015). Fruiting efficiency: An alternative trait to further rise wheat yield. Food Energy Secur..

[47] Gennaro A., Forte P., Carozza R., Savo Sardaro M.L., Ferri D., Bitti A., Borrelli G.M., D’Egidio M.G., Ceoloni C. (2007). Pyramiding different alien chromosome segments in durum wheat: Feasibility and breeding potential. Isr. J. Plant Sci..

[48] Valluru R., Reynolds M.P., Lafarge T. (2015). Food security through translational biology between wheat and rice. Food Energy Secur..

[49] Royo C., Álvaro F., Martos V., Ramdani A., Isidro J., Villegas D., García Del Moral L.F. (2007). Genetic changes in durum wheat yield components and associated traits in Italian and Spanish varieties during the 20th century. Euphytica.

[50] Reynolds M., Chapman S., Crespo-Herrera L., Molero G., Mondal S., Pequeno D.N.L., Pinto F., Pinera-Chavez F.J., Poland J., Rivera-Amado C. (2020). Breeder friendly phenotyping. Plant Sci..

[51] Reynolds M., Bonnett D., Chapman S.C., Furbank R.T., Manés Y., Mather D.E., Parry M.A.J. (2011). Raising yield potential of wheat. I. Overview of a consortium approach and breeding strategies. J. Exp. Bot..

[52] Furbank R.T., Jimenez-Berni J.A., George-Jaeggli B., Potgieter A.B., Deery D.M. (2019). Field crop phenomics: Enabling breeding for radiation use efficiency and biomass in cereal crops. New Phytol..

[53] Furbank R.T., Sharwood R., Estavillo G.M., Silva-Perez V., Condon A.G. (2020). Photons to food: Genetic improvement of cereal crop photosynthesis. J. Exp. Bot..

[54] Asseng S., Kassie B.T., Labra M.H., Amador C., Calderini D.F. (2017). Simulating the impact of source-sink manipulations in wheat. Field Crop. Res..

[55] Monsen S.B., Stevens R., Shaw N.L. (2004). Grasses. Restoring Western Ranges and Wildlands.

[56] Shu Y., Zhang J., Ao Y., Song L., Guo C. (2015). Analysis of the *Thinopyrum elongatum* transcriptome under water deficit stress. Int. J. Genomics.

[57] Ceoloni C., Kuzmanović L., Gennaro A., Forte P., Giorgi D., Grossi M.R., Bitti A., Tuberosa R., Graner A., Frison E. (2014). Genomes, chromosomes and genes of perennial triticeae of the genus *Thinopyrum*: The value of their transfer into wheat for gains in cytogenomic knowledge and ‘precision’ breeding. Advances in Genomics of Plant Genetic Resources.

[58] Li Z., Li B., Tong Y. (2008). The contribution of distant hybridization with decaploid *Agropyron elongatum* to wheat improvement in China. J. Genet. Genomics.

[59] Placido D.F., Campbell M.T., Folsom J.J., Cui X., Kruger G.R., Baenziger P.S., Walia H. (2013). Introgression of novel traits from a wild wheat relative improves drought adaptation in wheat. Plant Physiol..

[60] Placido D.F., Sandhu J., Sato S.J., Nersesian N., Quach T., Clemente T.E., Staswick P.E., Walia H. (2020). The LATERAL ROOT DENSITY gene regulates root growth during water stress in wheat. Plant Biotechnol. J..

[61] El Haddad N., Kabbaj H., Zaïm M., El Hassouni K., Tidiane Sall A., Azouz M., Ortiz R., Baum M., Amri A., Gamba F. (2021). Crop wild relatives in durum wheat breeding: Drift or thrift?. Crop Sci..

[62] Savary S., Willocquet L., Pethybridge S.J., Esker P., McRoberts N., Nelson A. (2019). The global burden of pathogens and pests on major food crops. Nat. Ecol. Evol..

[63] Alahmad S., Dinglasan E., Leung K.M., Riaz A., Derbal N., Voss-Fels K.P., Able J.A., Bassi F.M., Christopher J., Hickey L.T. (2018). Speed breeding for multiple quantitative traits in durum wheat. Plant Methods.

