# Supplementary material for: Small “Nested” Introgressions from Wild Thinopyrum Species, Conferring Effective Resistance to Fusarium Diseases, Positively Impact Durum Wheat Yield Potential
Source: Plants (Basel). 2021 Mar 19;10(3):579. doi: 10.3390/plants10030579 (PMC8003120; doi:10.3390/plants10030579)
Supplement: Supplementary file 1 [file plants-10-00579-s001.pdf]

Supplementary material

Small "nested" introgressions from wild *Thinopyrum* species conferring effective resistance to Fusarium diseases positively impact durum wheat yield potential

Kuzmanović L*, Giovenali G, Ruggeri R, Rossini F, Ceoloni C.

Table S1. Means ± standard errors of yield and yield-related traits recorded in 2019 and 2020 seasons for 7el$_1$L+7el$_2$L recombinants (R193+, R216+) and their null segregates (R193-, R216-) under rainfed growing conditions.

RL	GY (g)	TILN	GN	TGW (g)	GYS (g)	SPN	GNS	GNSP
**2019**								
R193+	17.5 ± 1.4	11.3 ± 0.9	354.7 ± 20.7	49.1 ± 1.9	3.01 ± 0.10	18.2 ± 0.3	53.0 ± 1.8	2.9 ± 0.1
R193-	18.1 ± 1.8	12.0 ± 1.9	416.9 ± 45.9	44.3 ± 2.4	3.05 ± 0.15	18.5 ± 0.4	57.7 ± 2.7	3.1 ± 0.1
R216+	11.8 ± 0.9	10.3 ± 0.9	268.8 ± 18.1	43.9 ± 2.2	2.20 ± 0.08	17.2 ± 0.3	41.7 ± 1.1	2.4 ± 0.04
R216-	13.6 ± 1.9	12.3 ± 1.1	315.9 ± 36.6	42.7 ± 1.7	2.69 ± 0.10	18.3 ± 0.3	52.4 ± 1.5	2.9 ± 0.1
**2020**								
R193+	10.3 ± 1.6	4.0 ± 0.5	203.1 ± 29.1	51.1 ± 2.7	2.46 ± 0.12	17.1 ± 0.3	46.3 ± 1.7	2.7 ± 0.1
R193-	10.9 ± 1.1	4.3 ± 0.4	208.6 ± 17.4	52.6 ± 3.0	2.89 ± 0.13	18.8 ± 0.3	53.8 ± 1.5	2.9 ± 0.1
R216+	10.8 ± 1.5	5.5 ± 0.7	208.1 ± 31.1	53.0 ± 1.3	2.45 ± 0.08	18.5 ± 0.2	45.8 ± 1.4	2.5 ± 0.1
R216-	9.6 ± 1.2	4.3 ± 0.4	164.1 ± 20.2	58.9 ± 0.9	2.39 ± 0.11	16.8 ± 0.4	40.2 ± 1.7	2.4 ± 0.1

GY, grain yield/plant; TILN, tiller number/plant; GN, grain number/plant; TGW, 1000-grain weight; GYS, grain yield/spike; SPN, spikelet No./spike; GNS, grain No./spike; GNSP, grain No./spikelet; RL, recombinant line

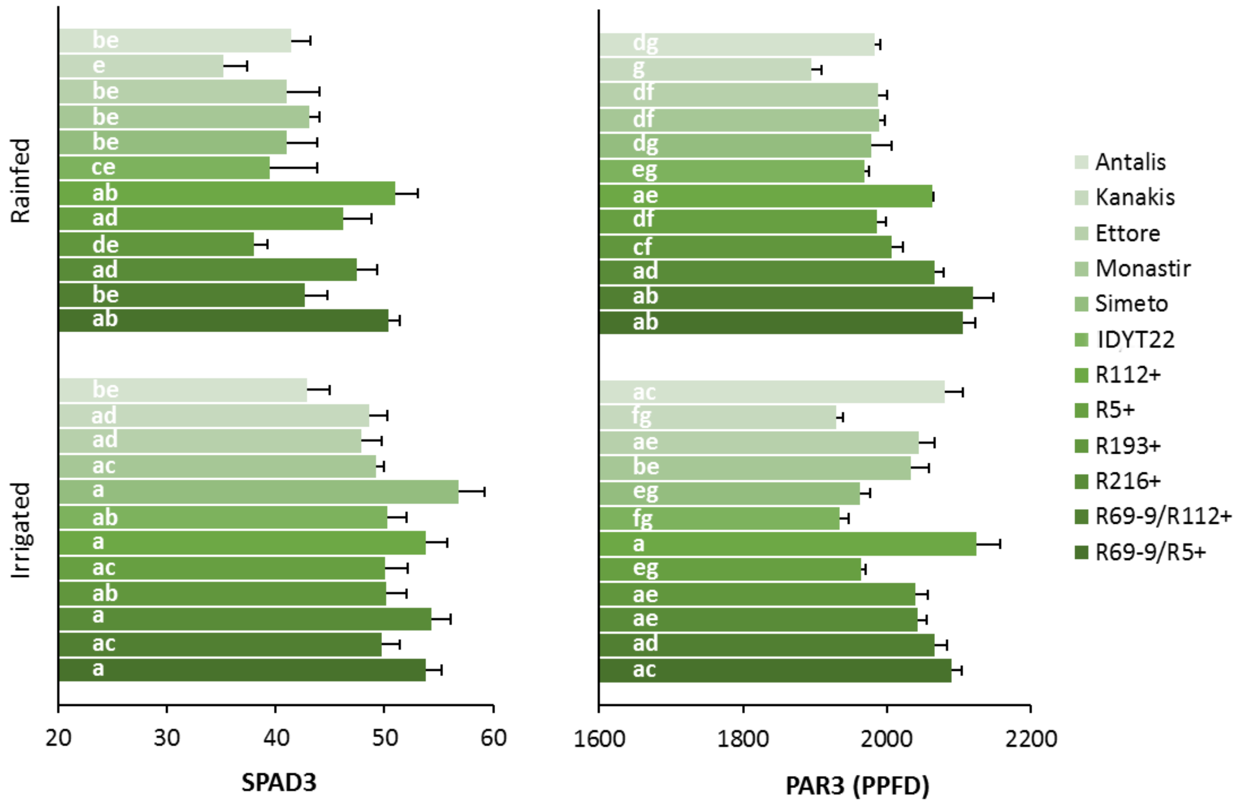

Figure S1. Significant Genotype × Irrigation effect on flag leaf physiological traits measured in 2020 under rainfed and irrigated conditions (SPAD3, chlorophyll content at 20 days post-anthesis; PAR3, photosynthetically active radiation of PSII at 20 days post-anthesis); bars represent standard errors of means; letters within histograms correspond to ranking of the Tukey test at $p < 0.05$ level.

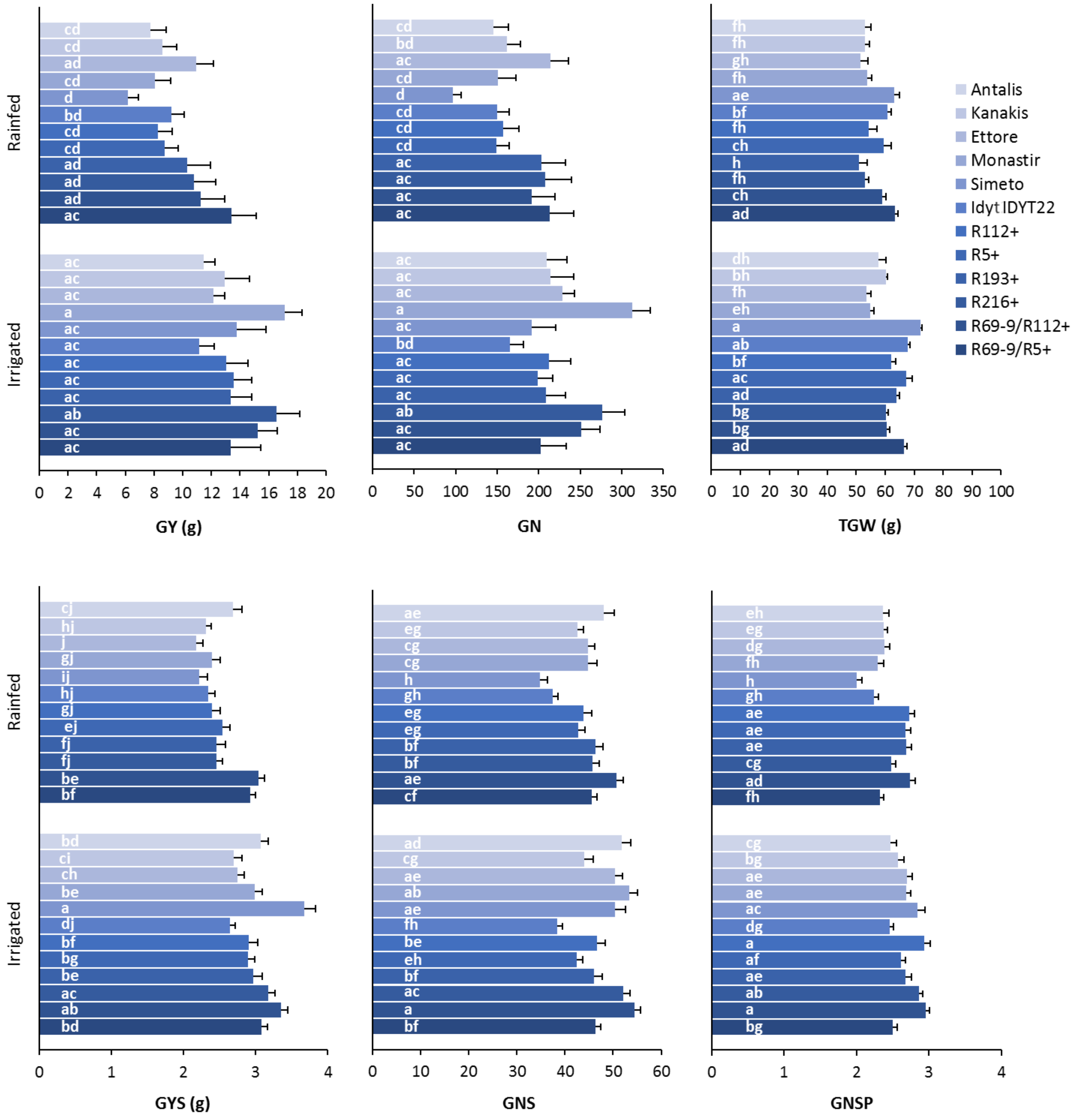

Figure S2. Significant Genotype × Irrigation effect on yield-related traits measured in 2020 under rainfed and irrigated conditions (GY, grain yield/plant; GN, grain number/plant; TGW, 1000-grain weight; GYS, grain yield/spike; GNS, grain No./spike; GNSP, grain No./spikelet; bars represent standard errors of means; letters within histograms correspond to ranking of the Tukey test at $p < 0.05$ level.
